# Evaluating the accuracy, quality, and reliability of septoplasty-related content on TikTok

**DOI:** 10.3389/fdgth.2026.1934459

**Published:** 2026-09-23

**Authors:** Raika Bourmand, Aayush Shah, Angel Sheu, Reshma Soundharrajan, Sofia Olsson, Viraj Shah

**Affiliations:** 1 Anne Burnett Marion School of Medicine, Texas Christian University, Fort Worth, TX, United States; 2 Department of Computer Science & Information Science & Engineering, University of Florida, Gainesville, FL, United States; 3 Department of Otolaryngology - Head and Neck Surgery, University of California Davis Medical Center, Sacramento, CA, United States; 4 Department of Otolaryngology - Head and Neck Surgery, University of Texas Southwestern, Dallas, TX, United States

**Keywords:** deviated septum, otolaryngology, septoplasty, social media, TikTok

## Abstract

**Introduction:**

TikTok has become a major source of health information, but concerns remain regarding the quality and reliability of medical content. Septoplasty is one of the most performed otolaryngologic procedures and is frequently discussed on the platform. We seek to evaluate the quality, reliability, and accuracy of septoplasty-related content on TikTok.

**Methods:**

A cross-sectional content analysis of publicly available TikTok videos was conducted between April and May 2025. Videos were identified using the search terms “septoplasty,” “deviated septum,” and “septal reconstruction.” After screening, 173 videos were included for analysis using the Global Quality Scale (GQS) and modified DISCERN (mDISCERN); 74 videos contained sufficient health information for evaluation with the Accuracy in Digital-health Instrument (ANDI). Descriptive and comparative analyses assessed content quality, reliability, accuracy, and engagement across creator types.

**Results:**

Overall, videos demonstrated moderate accuracy (mean ANDI 2.82), low-to-moderate quality (mean GQS 2.31), and low reliability (mean mDISCERN 0.78). Most videos (67.1%) were created by non–health care professionals. Physician-created videos had significantly higher accuracy, quality, and reliability scores than nonphysician videos (all *P* ≤ .001). Engagement metrics did not differ significantly by creator type (all *P* > .30). Verified account status was the factor most consistently associated with higher engagement.

**Conclusion:**

Septoplasty-related TikTok content demonstrates moderate accuracy but generally low reliability and quality. Content created by physicians and other healthcare professionals demonstrated higher accuracy, quality, and reliability than content created by non-healthcare professionals. These findings support greater participation by qualified healthcare professionals in disseminating evidence-based information on social media, although the effects of such content on viewer understanding and healthcare decision-making were not assessed.

## 1 Introduction

Social media has rapidly grown as a source of health-related information, influencing the way patients learn about various pathologies and treatments. According to a 2021 study, social media was considered a primary source of information during the COVID-19 pandemic (1). Nearly 80% of United States (US) health departments maintain social media accounts, including Facebook, underscoring the essential role of social media in contemporary public health communication (2). Among existing platforms, TikTok (3) stands out for its short-form videos, wide reach of users, and critical influence especially on younger audiences (4). The growth of the platform is noteworthy—expanding from 133 million users at its inception to over 1.7 billion in 2024—making it one of the world's leading social media platforms and highlighting its dominance relative to other networks (5). With TikTok's widespread reach and rapid growth, it increasingly serves as a space for medical advice and personal health experiences to be shared outside of traditional healthcare settings; for example, a recent study found that young women in the U.S. report obtaining both intentional and unintentional health information from TikTok (6). A recent systematic review assessing fifty different articles found that TikTok is widely used among patients from different surgical specialties and has an influence on their education, demonstrating its relevance in the surgical decision-making process (7).

Although this accessibility enhances patient engagement, it raises concerns for the accuracy and reliability of information online due to a lack of fact-checking of content.

These concerns are especially prevalent for surgical interventions such as septoplasty, given the elective nature of the procedure and the potential risks and complications—such as septal perforation, changes in sense of smell, bleeding, or infection—which make thorough informed consent and patient expectation management essential (8). Septoplasty is a surgery which may be performed endonasally, endoscopically, or open to remove a portion of the septal cartilage. The most common indication for this procedure is to correct septal deviation and relieve nasal airway obstruction (9, 10). Anatomical studies of human skulls suggest that septal deviation is highly prevalent, affecting approximately 75%–80% of individuals; however, only a small fraction of around 2% experience clinically significant symptoms related to the deviation, underscoring the distinction between anatomical presence and symptomatic relevance (11–13). Due to the prevalence of septal deviation, septoplasty is one of the most commonly performed procedures within otolaryngology specialties (9). While the procedure is generally safe and effective (14), misconceptions may exist about risks of surgery, recovery time and outcomes. Additionally, patients online may not distinguish between septoplasty and other nasal surgeries such as septorhinoplasty which present with different risk profiles and recovery. Inaccurate or oversimplified online content may raise concerns regarding the potential for misinformation, misunderstanding, or unrealistic expectations about the procedure. To objectively evaluate the quality of online health information, researchers have used validated tools such as the Global Quality Score (GQS) and modified DISCERN (mDISCERN) (15). The GQS scale rates overall quality, flow, and usefulness of health information, with higher scores indicating superior quality (16). The mDISCERN scale, adapted from the original DISCERN instrument, assesses the reliability and trustworthiness of health content, including clarity, sources, and balance (15).

These instruments have been applied across various specialties and online platforms. For instance, previous studies have evaluated TikTok content on hypertension, hysteroscopy, and orthognathic surgery using GQS and mDISCERN, demonstrating their versatility to evaluate clarity, reliability and educational value, allowing for a comprehensive analysis of qualitative patient-facing information (17–19).

The purpose of this study is to systematically evaluate septoplasty-related TikTok videos for their quality, reliability, and accuracy. By applying structured assessment tools including the GQS, ANDI, and mDISCERN, this study aims to characterize the quality and accuracy of septoplasty-related content on TikTok, examine differences according to creator and content characteristics, and identify opportunities to improve the dissemination of evidence-based health information in the digital space.

## 2 Methods

Institutional review board approval was not sought for this study, as it analyzed publicly available, non-identifiable data in accordance with Institutional Review Board guidelines.

From April 30, 2025 through May 3, 2025, two independent researchers systematically queried the terms “*septoplasty*,” “*deviated septum*,” and “*septal reconstruction*” on TikTok. These terms were selected to capture both formal surgical terminology and colloquial language that patients and the general public are likely to use, ensuring inclusion of videos from creators with varying expertise and perspectives. Additionally, the three terms were chosen to encompass all aspects of nasal septum surgery while excluding unrelated rhinologic procedures to prevent procedure-specific bias in the results.

To prevent algorithmic personalization, newly created TikTok accounts with no prior history were used. For each search term, the first 50 videos returned by TikTok were collected to capture content prominently surfaced to users at the time of the search while maintaining a standardized sampling approach across search terms. Searches were conducted using fresh TikTok accounts to minimize the influence of prior account activity and personalized viewing history on search results. Investigators were instructed to collect the 50 videos for each search term in a single session when possible and to exclude unrelated sponsored content. Because TikTok search results remain algorithmically generated and may vary according to temporal trends, platform activity, and other proprietary factors, this approach was intended to characterize prominently surfaced content rather than to generate a random or exhaustive sample of all septoplasty-related videos.

After excluding duplicates and videos unrelated to septoplasty from the initial collection, 173 unique videos remained, comprising the dataset for analysis. Data collection procedures were informed by previously published studies evaluating medical content on social media (20–24). A custom Python script was developed to extract video metadata, including engagement metrics (views, likes, shares, and comments), video duration, upload timestamp, creator credentials, and indicators of paid promotion.

A standardized codebook, available in Supplementary Table S1, was developed by the research team prior to analysis in order to maximize consistent categorization of video content. Videos were classified as one or more of the following purpose codes: educational, anecdotal, or entertainment-focused, with operational definitions, inclusion/exclusion criteria, and illustrative examples for each category. Multiple purpose codes could be assigned to a single video if content met criteria for more than one category.

Three scoring instruments were used to evaluate the quality, reliability, and accuracy of health information presented in the dataset. The Global Quality Scale (GQS), with a range of 1–5 (Supplementary Table S2) provides an overall measure of the quality, flow, and educational value of videos, with a score of 1 indicating poor quality and 5 indicating excellent quality (15). The modified DISCERN scale (mDISCERN), with a range of 0–5 (Supplementary Table S3), evaluates the reliability of health information using five criteria, with one point awarded for each criterion satisfied and higher scores indicating greater reliability (15). The Accuracy in Digital-Health Instrument scale (ANDI), with a 0–4 score (Supplementary Table S4) was used to determine factual correctness based on current clinical guidelines for septoplasty, including indications, surgical approaches, management of deviated septum, and postoperative expectations (25). For ANDI, a score of 0 denoted completely inaccurate information, while a score of 4 signified fully accurate information. Information was described as accurate based on the current septoplasty guidelines including initial evaluation or management of conditions like nasal airway obstruction, frequent nosebleeds, or atypical facial pain (25). ANDI scoring was performed only for videos containing sufficient substantive health information to permit an assessment of factual accuracy. Videos were considered eligible for ANDI scoring if they contained sufficient information related to septoplasty, including information regarding indications, evaluation or management of relevant symptoms, surgical treatment, or postoperative expectations. Videos without sufficient clinical information to permit an accuracy determination were not assigned an ANDI score but remained included in all other applicable analyses. These instruments have been used in prior studies assessing online medical content (16, 26, 27). Each eligible video was independently rated by two researchers. When ratings differed, no single rater's score was preferentially selected; instead, the mean of the two independent ratings was used for the final analyses. Inter-rater reliability for ANDI, GQS, and mDISCERN scores was calculated using quadratic-weighted Cohen's kappa to account for the ordinal nature of the scales.

Creators were classified into two main categories: healthcare professionals (HCPs) and non-healthcare professionals (non-HCPs). An HCP was defined as any individual holding verifiable clinical qualifications. Within the HCP group, a distinction was made between physicians (individuals with an MD or DO degree) and other allied HCPs, which included nurses, physician assistants, and other licensed non-physician practitioners. The identification of septoplasty-related content followed a two-step approach. Initially, researchers systematically documented all relevant qualitative clinical information presented in each video, including patient symptoms, surgical techniques, and postoperative care recommendations. These records were subsequently processed, and duplicates were removed using custom Python scripts to produce accurate counts of content occurrences for further analysis.

Statistical analyses were conducted using Python 3.11.10 with statsmodels, scipy, pandas, and numpy. Descriptive statistics (counts, means, standard deviations, medians, ranges, frequencies, and proportions) were calculated for all variables. Mann–Whitney *U*-tests were used to compare quality metrics based on content purpose and creator type because of the ordinal and/or non-normally distributed nature of the outcomes. Pearson correlation coefficients were used to assess linear relationships between quality scores, log-transformed engagement metrics, and video duration, while Spearman rank correlation coefficients were used to assess monotonic relationships between quality scores and creator profile metrics. Multiple linear regression models were used to evaluate associations between video and creator characteristics and log-transformed engagement outcomes. Engagement metrics were log-transformed because of their highly right-skewed distributions. Predictor variables were selected based on their relevance to the study objectives and included video quality scores, video duration, creator characteristics, and content purpose. Given the sample size, multivariable analyses were considered exploratory. To account for multiple comparisons, *P* values were adjusted using the Benjamini-Hochberg false discovery rate procedure where applicable. Model diagnostics included assessment of residual distributions, heteroscedasticity, multicollinearity, and influential observations. Residual distributions and residual-vs.-fitted behavior were examined to assess linear model assumptions, and variance inflation factors were used to assess multicollinearity. Statistical significance was defined as *P* ≤ .05.

A sensitivity analysis was performed to assess whether the primary creator-type findings were robust after excluding videos containing cosmetic content, which could represent mixed septoplasty/rhinoplasty or septorhinoplasty content. The sensitivity analysis was restricted to videos without coded cosmetic mentions, and HCP vs. non-HCP differences in ANDI, GQS, and mDISCERN were reassessed using Mann–Whitney *U*-tests with Benjamini-Hochberg correction for multiple comparisons.

## 3 Results

### 3.1 Video characteristics and creator demographics

A total of 173 unique videos discussing septoplasty were included in the study. Of these, 74 videos contained sufficient substantive health information to permit assessment of factual accuracy using ANDI. The remaining 99 videos did not contain sufficient assessable clinical information and therefore were not assigned an ANDI score but remained included in all other applicable analyses. Among the 74 videos eligible for ANDI scoring, 33 (44.6%) were created by healthcare professionals and 41 (55.4%) by non-healthcare professionals. Accuracy as measured by ANDI had a mean of 2.82 (SD 1.21), indicating moderate to good accuracy (Table 1). Video quality measured by GQS scored a mean of 2.31 (SD 1.18), reflecting low to moderate quality (Table 1). Reliability measured by mDISCERN scored a mean of 0.78 (SD 0.74), indicating low reliability (Table 1).

**Table 1:** Descriptive statistics of video quality and engagement metrics.

Measure	Count	Mean (SD)	Median	IQR	Range
Quality Scores					
ANDI	74	2.82 (1.21)	3	2.50–4.00	0.00–4.00
GQS	173	2.31 (1.18)	2	1.00–3.00	0.50–5.00
mDISCERN	173	0.78 (0.74)	1	0.00–1.00	0.00–3.00
Engagement Metrics					
Views	173	640,490 (2,243,986)	46,700	11,300–226,200	98–19,400,000
Likes	173	35,011 (135,826)	975	178–5,256	2–1,000,000
Shares	173	1,236 (4,588)	49	8–270	0–33,600
Comments	173	285 (1,208)	39	12–135	0–12,400

Video engagement was highly variable, with the median number of views per video being 46,700 (IQR 11,300–226,200) (Table 1). When viewing a video on TikTok, users have the option to “like” the post. The median number of likes per septoplasty-related video was 975 (IQR 178–5,256) (Table 1). Users may also “share” videos to others. Videos in this sample had an average of 49 (IQR 8–270) shares per video and 39 (IQR 12–135) comments per video (Table 1). The median video duration was 27.0 s (IQR 13.0–63.0).

Non–HCPs produced 67.1% (*n* = 116) of videos, physicians 17.9% (*n* = 31), and other healthcare professionals 15.0% (*n* = 26). Creators were predominantly female (55.5%), followed by male (26.0%), and the gender of the remaining 18.5% could not be determined.

Content analysis determined that 47.4% of videos were educational in nature, 42.8% entertainment-focused, and 73.4% anecdotal as defined in Supplementary Table S1. Appearance of the nose and cosmetic mentions occurred in 31.2% of videos which may indicate that despite our septoplasty-focused search terms, videos discuss septoplasty, rhinoplasty, and septorhinoplasty, often without distinguishing between the procedures. Analysis of video content showed that 42.2% of videos incorporated trending topics (defined as the use of popular sounds, challenges, or other TikTok trends), and 79.8% utilized visual elements (defined as supplementary graphics like on-screen text, images, charts, or diagrams). Paid promotional content was rare (1.2%).

The type of content produced differed significantly by creator category. Healthcare professionals were significantly more likely to create educational content than non-HCPs (73.1% of other HCPs and 64.5% of physicians vs. 37.1% of non-HCPs; *P* < 0.001). Conversely, non-HCPs were more likely to produce entertainment-focused content (50.0%) compared to physicians (35.5%) and other HCPs (19.2%; *P* = 0.011). Anecdotal content was also most prevalent among non-HCPs, appearing in 87.1% of their videos, compared to 46.2% for other HCPs and 45.2% for physicians (*P* < 0.001).

### 3.2 Inter-Rater reliability

Quadratic-weighted Cohen's kappa values were 0.501 for ANDI (moderate agreement, *n* = 74), 0.973 for GQS (excellent agreement), and 0.808 for mDISCERN (excellent agreement).

### 3.3 Associations with video quality

Creator type was significantly associated with accuracy, quality, and reliability after Benjamini–Hochberg correction (Table 2). HCPs scored higher than non-HCPs professionals across all measures: ANDI (mean 3.11 vs. 2.60, *P* = 0.001), GQS (mean 2.78 vs. 2.08, *P* < 0.001), and mDISCERN (mean 1.20 vs. 0.58, *P* < 0.001) (Figure 1). Physicians (MD/DO) in particular had the highest scores across all instruments, with significantly greater ANDI, GQS, and mDISCERN compared with non–healthcare professionals (all *P* ≤ 0.001): ANDI (mean 3.73 vs. 2.60), GQS (mean 2.98 vs. 2.08), and mDISCERN (mean 1.29 vs. 0.58). Compared with other HCPs (non-physician HCPs), physicians achieved higher ANDI scores (mean 3.73 vs. 2.15, *P* = 0.017) indicating greater accuracy, whereas differences in GQS (mean 2.98 vs. 2.54) and mDISCERN (mean 1.29 vs. 1.10) were not significant after correction (Table 2).

**Table 2:** Comparison of video quality scores by creator type.

Metric (Group 1 vs. Group 2)	Group 1 Mean	Group 2 Mean	*p*-value
HCP vs. Non-HCP			
ANDI	3.11	2.6	0.001
GQS	2.78	2.08	<0.001
mDISCERN	1.2	0.58	<0.001
MD/DO vs. Non-HCP			
ANDI	3.73	2.6	<0.001
GQS	2.98	2.08	<0.001
mDISCERN	1.29	0.58	<0.001
MD/DO vs. Other HCP			
ANDI	3.73	2.15	0.017
GQS	2.98	2.54	0.195
mDISCERN	1.29	1.1	0.255
Other HCP vs. Non-HCP			
ANDI	2.15	2.6	0.910
GQS	2.54	2.08	0.028
mDISCERN	1.1	0.58	0.003

Mann–Whitney *U-*test was used for comparisons. Corrected *p*-values are reported, and significant values (*p* < 0.05) are bolded. HCP, Healthcare Professional. Other HCP refers to HCP's that are not MD/DO.

**Figure 1:**
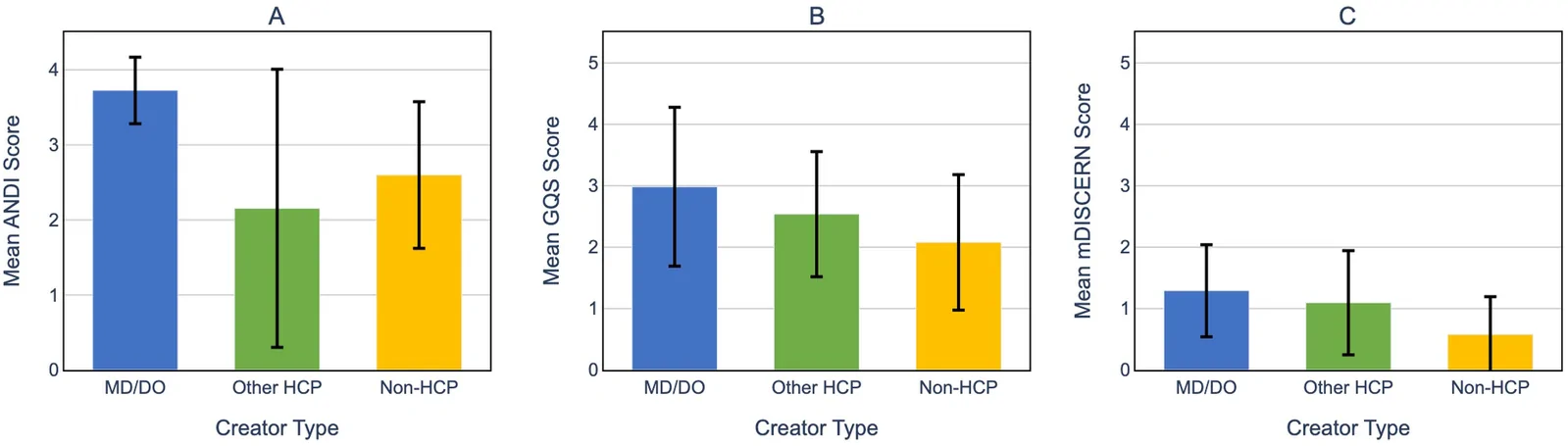
**(A)** mean aNDI score by creator type **(B)** mean GQS by creator type **(C)** mean mDISCERN by creator type.

In contrast to the findings on video quality, there were no significant differences in engagement metrics based on creator type. Median views, likes, shares, and comments were not significantly different between videos created by physicians, other HCPs, and non-HCPs (all *p* > .30).

Content purpose significantly influenced accuracy, quality, and reliability. Educational videos had significantly higher ANDI (mean 2.93 vs. 2.44, *P* = 0.021), GQS (mean 3.16 vs. 1.54, *P* < 0.001), and mDISCERN (mean 1.29 vs. 0.32, *P* < 0.001) scores than non-educational content. Conversely, entertainment and anecdotal content were associated with lower GQS and mDISCERN scores (all *P* < 0.001).

Creator gender also demonstrated significant associations with video quality metrics. Kruskal–Wallis tests showed significant differences in GQS (H = 11.943, *P* = 0.008) and mDISCERN (H = 17.012, *P* = 0.001). Video creators who did not show their face had the highest GQS and mDISCERN scores, whereas male and female creators showed similar distributions.

### 3.4 Content features and quality associations

Use of trending topics was significantly associated with lower quality and reliability. Videos without trending topics achieved higher GQS (mean 2.69 vs. 1.79; corrected *P* < 0.001) and mDISCERN (mean 0.97 vs. 0.53; corrected *P* < 0.001) scores compared with those incorporating trends. Within subgroups, both physicians and non–healthcare professionals demonstrated significantly lower GQS and mDISCERN when using trends.

Cosmetic mentions were most frequent among physician creators (41.9%) and often reflected rhinoplasty content despite a septoplasty query. Cosmetic mentions were associated with higher ANDI scores among non–healthcare professionals and lower mDISCERN scores among other healthcare professionals, though these associations did not remain significant after correction. Visual elements were common across creator types, particularly among healthcare professionals, but showed no significant association with quality measures.

### 3.5 Content features and engagement

Videos mentioning cosmetic aspects of septoplasty demonstrated significantly lower engagement across views (median 30,000 vs. 91,000, *P* = 0.004), likes (433 vs. 1,375, *P* = 0.015), and shares (20 vs. 94, *P* = 0.001).

Verified creators demonstrated substantially higher engagement, with median view counts 24-fold higher than non-verified creators (932,100 vs. 38,200, *P* < 0.001), along with significantly higher likes (10,300 vs. 628, *P* < 0.001) and comments (191 vs. 32, *P* < 0.001). Verified creators also produced videos with higher ANDI (mean 3.62 vs. 2.66, *P* = 0.014) and GQS (mean 3.00 vs. 2.22, *P* = 0.023) scores.

### 3.6 Predictors of video engagement

Regression models explained between 16.8% and 32.1% of variance in engagement outcomes. Author verification was the most consistent factor associated with engagement, with verification status significantly associated with higher view counts (*β* = 1.87, *P* = 0.015), likes (*β*=2.45, *P* = 0.007), and comments (*β* = 1.42, *P* = 0.050). Entertainment content predicted higher like counts (*β* = 2.30, *P* = 0.033). Individual quality scores (ANDI, GQS, mDISCERN) and other content characteristics were not significant predictors. These findings indicate that platform recognition and entertainment value were more strongly associated with engagement than measures of content quality in this sample. Model diagnostics did not identify substantial violations of residual distribution or heteroscedasticity assumptions, and variance inflation factors did not indicate severe multicollinearity; however, potentially influential observations were present and the regression findings were therefore interpreted as exploratory.

### 3.7 Sensitivity analysis

Of the 173 videos, 54 (31.2%) contained cosmetic mentions and were excluded from the sensitivity analysis, leaving 119 videos without cosmetic content. Among this restricted sample, HCP-created videos continued to demonstrate significantly higher GQS (mean 2.99 vs. 2.14; adjusted *P* < 0.001) and mDISCERN (mean 1.33 vs. 0.59; adjusted *P* < 0.001) scores than non-HCP-created videos. Among the 62 videos in the restricted sample eligible for ANDI assessment, HCP-created videos also demonstrated significantly higher ANDI scores (mean 3.00 vs. 2.44; adjusted *P* = 0.003). These findings were consistent with the primary analysis.

## 4 Discussion

This study critically evaluated septoplasty-related content on TikTok, revealing a concerning mismatch between the quality of information and audience engagement. While we found that videos achieved moderate accuracy (mean ANDI 2.82) and a low to moderate overall quality (mean GQS 2.31), reliability of the content remained poor (mean mDISCERN 0.78). In the context of mDISCERN, such low scores indicate that most videos failed to cite sources, reference evidence-based information, or disclose potential biases, limiting their trustworthiness despite moderate accuracy and quality (15). These findings demonstrate a consistent problem that health-related content on social media, although widely consumed, is not consistently trustworthy (28, 29).

We found that a key determinant of content quality was creator type. The results of this study revealed that physicians produced the most accurate, reliable and higher-quality content, which outperformed both non-healthcare professionals and other healthcare providers. This mirrors results from Shah et al., who showed that educational hyperthyroidism-videos (98.3% of their sample) had higher GQS and mDISCERN scores than non-educational content, and that anecdotal videos were associated with significantly lower quality and reliability (30, 31). Despite this, physicians represented even less than one-fifth of creators, and their videos included cosmetic themes which diluted educational focus. This finding raises questions about the different professional motivations which can be found as a result of the wide reach of the TikTok platform, when comparing the intent of educating vs. marketing. The frequent overlap between cosmetic and functional content may make it more difficult for viewers to distinguish between septoplasty, rhinoplasty, and septorhinoplasty (31, 32). However, the present study did not assess viewer understanding or determine whether exposure to such content resulted in misinformation or changes in healthcare decision-making.

Although healthcare professionals demonstrated higher accuracy, engagement was more strongly associated with platform-related characteristics, including verification status and entertainment content. Verified creators had substantially higher engagement than unverified creators; however, given the cross-sectional observational design, this association should not be interpreted as evidence that verification itself causes increased engagement. Verification may instead reflect other creator characteristics, such as established audience size, visibility, or platform prominence. This aligns with findings by Neely et al. (33), who found that verification badges significantly increase users' perceptions of the accuracy of posted information (33). Similarly, in the coronary artery disease videos study by Hao et al., medical and organizational creators (who are more likely to be recognized/verified) scored higher on quality metrics, suggesting that source recognition continues to influence perceptions of credibility and quality (34). Importantly, TikTok verification is generally restricted to users who are widely recognized or appear in the news, meaning that most physicians—especially those not in high-profile media—cannot easily obtain verification (35). This creates a structural bias in favor of already visible creators and highlights that even highly accurate healthcare professionals may struggle to achieve reach unless they strategically adapt to the platform without compromising educational value. For example, a verified social media influencer with minimal medical training may achieve far greater engagement than a non-verified physician posting highly accurate septoplasty content. The persistence of these differences in a sensitivity analysis excluding videos with cosmetic content suggests that the association between healthcare professional creator status and higher content quality was not solely attributable to the inclusion of mixed rhinoplasty or cosmetic content.

Trending topics, while central to the culture of TikTok, were also strongly associated with lower quality and reliability. This association suggests that trend-based content in our sample tended to demonstrate lower overall quality and reliability. Prior studies of plastic surgery and dermatology content on TikTok have also noted that trend-driven videos prioritize visibility over depth and reliability (36, 37). On a similar note, cosmetic mentions were linked to reduced engagement, challenging the assumption that framing content with the theme of aesthetics drives popularity. This observation aligns with prior research suggesting that social media content emphasizing cosmetic procedures does not necessarily generate greater engagement and may even limit audience reach, particularly when it highlights unrealistic aesthetic standards (38). These patterns suggest that while users value entertainment and platform recognition, they do not necessarily reward cosmetic framing when seeking health information.

Creators with non-identifiable gender, such as videos without a visible presenter, achieved the highest quality scores, while male and female creators showed similar distributions. Previous analyses of health communication on social media suggest that depersonalized or infographic style content can outperform personality-driven content in clarity and reliability (30, 39). Future work is encouraged to examine if such formats promote clarity and reduce biases in health communication.

Lastly, regression models accounted for at most approximately one-third of the variance in engagement, indicating that substantial variation was not explained by the measured creator, content, and quality characteristics. Notably, video quality scores—including accuracy, reliability, and overall educational value—were not significantly associated with engagement outcomes in the multivariable models. Prior studies have similarly reported discordance between the quality of online health information and its engagement or visibility (40, 41). Collectively, these findings suggest that content quality alone may not account for the dissemination and engagement of health information on digital platforms (40, 41).

Clinically, these dynamics have important implications for ENT practice. Patients seeking information on septoplasty via TikTok may encounter anecdotal, entertainment-focused, or low-reliability content regarding surgical indications, expected outcomes, and recovery. Although physician-created content is more accurate, limited visibility underscores the need for ENT surgeons to strategically engage with social media. Verified creators demonstrated markedly higher engagement and higher quality scores, suggesting that creator characteristics and platform visibility may be important considerations in the dissemination of evidence-based health information on social media. However, this study did not assess whether these characteristics influenced viewer understanding or subsequent healthcare decision-making.

Recent work has also highlighted the potential value of procedure-specific instruments for evaluating surgical video content. Derin et al. evaluated videos of inferior alveolar nerve repositioning using both general quality measures and a procedure-specific technical index incorporating critical operative steps, demonstrating the potential for targeted instruments to provide a more granular assessment of surgical video content than general quality scales alone (42). Although the present study incorporated ANDI to assess factual accuracy in addition to GQS and mDISCERN, ANDI is not specific to septoplasty technique. Future studies may therefore benefit from the development of a septoplasty-specific assessment instrument incorporating relevant procedural elements, such as indications, operative principles, risks, postoperative care, and expected outcomes.

Although this study focused specifically on TikTok, other social media platforms, including YouTube, Instagram, and Facebook, also serve as sources of health information and may differ in content format, audience characteristics, search functionality, and mechanisms of content dissemination. TikTok was selected because its short-form, algorithmically distributed video format provides a distinct environment for health information sharing and engagement. Accordingly, the present findings should not be assumed to generalize to other social media platforms. Future studies directly comparing septoplasty-related content across platforms may help determine whether differences in accuracy, quality, and reliability are platform-specific. Potential strategies to improve the quality of online surgical information include greater participation by qualified healthcare professionals, development of concise and platform-appropriate evidence-based educational content, and clearer communication of creator credentials and information sources. Collaboration among healthcare professionals, professional societies, and social media platforms may also provide opportunities to improve the visibility and accessibility of reliable health information. Future research should evaluate whether such interventions improve the quality of information encountered by users and, importantly, whether they improve viewer comprehension or healthcare decision-making.

### 4.1 Limitations and future directions

The present study was restricted to a single platform and a limited set of procedure-specific search terms—“septoplasty,” “deviated septum,” and “septal reconstruction”—potentially overlooking other relevant terminology or related procedures, which may limit generalizability to broader ENT content or social media platforms. Although validated instruments were used, assessment of video quality, reliability, and accuracy inevitably involves some degree of rater judgment. Inter-rater agreement was excellent for GQS and mDISCERN but moderate for ANDI (*κ* = 0.501), indicating greater rater-dependent variability in the assessment of factual accuracy. This may reflect the greater interpretive complexity involved in evaluating the accuracy and clinical context of individual health claims compared with broader assessments of overall quality and reliability. Although the mean of two independent ratings was used to reduce reliance on any single rater's assessment, the moderate agreement for ANDI introduces additional measurement uncertainty and should be considered when interpreting accuracy-related findings. Additionally, ANDI could only be applied to the 74 videos containing sufficient substantive health information to permit assessment of factual accuracy. Because videos lacking assessable clinical information were not assigned an ANDI score, the accuracy findings reflect this informative subset rather than the entire sample of 173 videos. This may introduce selection bias if videos containing assessable clinical claims differ systematically from videos without such content, and the ANDI findings should therefore not be generalized to all septoplasty-related videos in the sample.

Although multiple subgroup analyses, correlation assessments, and multivariable regression models were performed, some creator categories, particularly physicians and other healthcare professionals, contained relatively small sample sizes. As a result, certain analyses may have been underpowered, and regression models incorporating multiple predictors may be susceptible to instability, particularly given the skewed distribution of social media engagement metrics. The overall sample size and the smaller sizes of some creator subgroups may also limit the statistical power and precision of subgroup comparisons and multivariable analyses. Although false discovery rate correction was applied to account for multiple comparisons, findings from analyses involving smaller subgroups may be less stable and should be interpreted as exploratory. Larger studies are needed to confirm these associations across creator categories.

Engagement metrics such as views, likes, shares, and comments reflect exposure and interaction but do not capture whether viewers acquired accurate knowledge or made informed healthcare decisions. Additionally, because videos were collected between April and May 2025, the findings represent a cross-sectional snapshot of septoplasty-related content surfaced on TikTok during the study period. Although fresh TikTok accounts were used to minimize the influence of prior account activity and personalized viewing history, TikTok's search algorithm is dynamic and may be influenced by temporal trends, platform activity, and other proprietary factors. Consequently, the sampled videos may not be representative of all septoplasty-related content available on TikTok, and identical searches performed at another time or by another user may yield different results. The findings should therefore not be interpreted as representing the current or persistent state of septoplasty-related content on the platform. Furthermore, the cross-sectional observational design precludes causal inference regarding associations between creator or content characteristics and engagement. For example, the association between verification status and engagement may reflect unmeasured creator characteristics, such as established audience size, visibility, or platform prominence, rather than an effect of verification itself. Finally, this study did not assess viewer comprehension, perceptions, subsequent healthcare decisions, or other behavioral outcomes; therefore, the findings cannot determine whether exposure to the evaluated content resulted in patient misunderstanding, misinformation, altered expectations, or changes in healthcare-seeking behavior. Longitudinal or repeated cross-sectional studies are needed to evaluate how septoplasty-related content and its visibility change over time.

In addition, creator classification was based on publicly available TikTok profile information. Because professional credentials may be incomplete, difficult to verify, or inaccurately represented online, some degree of misclassification of healthcare professional status is possible. Although efforts were made to verify creator backgrounds whenever feasible, the accuracy of self-reported credentials could not be independently confirmed in all cases.

Future research should expand search strategies to include a wider range of procedure- and symptom-related terms, analyze other social media platforms, and incorporate qualitative assessments of viewer interpretation. Studies should investigate how social media content influences patient decision-making and evaluate interventions to improve the visibility and reach of credible surgical content. Future studies should also evaluate whether partnerships between healthcare professionals and influential creators or other platform-adapted communication strategies can improve the visibility and accessibility of reliable patient-facing ENT content.

## 5 Conclusion

Septoplasty-related TikTok content demonstrated variable accuracy, quality, and reliability, with healthcare professional-created content generally receiving higher scores than content produced by non-healthcare professionals. Engagement, however, was more strongly associated with creator and platform-related characteristics than with measures of content quality. These findings characterize the information environment encountered by users searching for septoplasty-related content on TikTok but do not establish how viewers interpret this information or whether it influences subsequent healthcare decisions. Future studies should directly evaluate viewer comprehension, perceptions, and behavioral responses to social media content related to septoplasty.

## Data Availability

Publicly available datasets were analyzed in this study. This data can be found here: https://docs.google.com/spreadsheets/d/1oeE5rMzrd4RrBsxM1SDAYv3FsCDpKCRIGSZ_mPxuQao/edit?usp=sharing.
