# Supplementary material for: Evaluating the accuracy, quality, and reliability of septoplasty-related content on TikTok
Source: Front Digit Health. 2026 Sep 23;8:1934459. doi: 10.3389/fdgth.2026.1934459 (PMC13642820; doi:10.3389/fdgth.2026.1934459)
Supplement: Supplementary file 1 [file Table1.docx]

**Supplemental Table 1: Coding Definitions for Video Quality Assessment**

| **Code (Content Purpose)** | **Definition** | **Example** |
| --- | --- | --- |
| Educational | Explains information relating to septoplasty, aiming to educate the audience. This includes content that discusses symptoms, treatments, causes, prevention, or management strategies in an informative manner. | A medical professional explains septoplasty: detailing key symptoms (e.g., difficulty breathing), common causes (e.g., deviated septum), and treatment options (e.g., medication, surgery). |
| Entertainment | Content mentioning septoplasty-related topics with the primary goal of entertaining the audience. This can include humorous takes, celebrity stories, or creative narratives where septoplasty is part of the storyline but not the central focus of education or factual dissemination. | An influencer creates a comedic skit exaggerating septoplasty symptoms like nasal obstruction, snoring, difficulty breathing, etc. |
| Anecdotal | Contains personal stories, experiences, or testimonies about septoplasty, where the primary focus is on sharing individual accounts rather than providing generalized information or aiming for entertainment. | An individual recounts their personal septoplasty story: covering their diagnosis process, initial symptoms (e.g., difficulty breathing), emotional impact, and treatment experiences. |
