# Supplementary material for: Evaluating the accuracy, quality, and reliability of septoplasty-related content on TikTok
Source: Front Digit Health. 2026 Sep 23;8:1934459. doi: 10.3389/fdgth.2026.1934459 (PMC13642820; doi:10.3389/fdgth.2026.1934459)
Supplement: Supplementary file 2 [file Table2.docx]

**Supplemental Table 2: Global Quality Scale.**

1 - Poor quality, poor flow, most information missing, not at all useful for patients

2 - Generally poor quality and poor flow, some information listed but many important topics missing, of very limited use to patients

3 - Moderate quality, suboptimal flow, some important information is adequately discussed but others poorly discussed, somewhat useful for patients

4 - Good quality and generally good flow, most of the relevant information is listed, but some topics not covered, useful for patients

5 - Excellent quality and excellent flow, very useful for patients
