# Supplementary material for: Evaluating the accuracy, quality, and reliability of septoplasty-related content on TikTok
Source: Front Digit Health. 2026 Sep 23;8:1934459. doi: 10.3389/fdgth.2026.1934459 (PMC13642820; doi:10.3389/fdgth.2026.1934459)
Supplement: Supplementary file 3 [file Table3.docx]

**Supplemental Table 3: Modified DISCERN Instrument.**

0 for no, 1 for yes Are the aims clear and achieved?

0 for no, 1 for yes Are reliable information sources used?

0 for no, 1 for yes Is the information balanced and unbiased?

0 for no, 1 for yes Are additional sources listed for patients?

0 for no, 1 for yes Are areas of uncertainty stated?

Sum of all 5 categories is the score 0-5.
