# Supplementary material for: Evaluating the accuracy, quality, and reliability of septoplasty-related content on TikTok
Source: Front Digit Health. 2026 Sep 23;8:1934459. doi: 10.3389/fdgth.2026.1934459 (PMC13642820; doi:10.3389/fdgth.2026.1934459)
Supplement: Supplementary file 4 [file Table4.docx]

**Supplemental Table 4: Accuracy in Digital-health Instrument (ANDI).**

0 - Completely inaccurate

1 - Minimally accurate

2 - Fairly accurate

3 - Mostly accurate

4 - Completely accurate
